# Vulnerability of Human Cerebellar Neurons to Degeneration in Ataxia-Causing Channelopathies

**DOI:** 10.3389/fnsys.2022.908569

**Published:** 2022-06-09

**Authors:** David D. Bushart, Vikram G. Shakkottai

**Affiliations:** ^1^Ohio State University College of Medicine, Columbus, OH, United States; ^2^Department of Neurology, University of Texas Southwestern Medical Center, Dallas, TX, United States

**Keywords:** ion channel, channelopathies, ataxia and cerebellar disorders, neurodegeneration, Purkinje cell

## Abstract

Mutations in ion channel genes underlie a number of human neurological diseases. Historically, human mutations in ion channel genes, the so-called channelopathies, have been identified to cause episodic disorders. In the last decade, however, mutations in ion channel genes have been demonstrated to result in progressive neurodegenerative and neurodevelopmental disorders in humans, particularly with ion channels that are enriched in the cerebellum. This was unexpected given prior rodent ion channel knock-out models that almost never display neurodegeneration. Human ataxia-causing channelopathies that result in even haploinsufficiency can result in cerebellar atrophy and cerebellar Purkinje neuron loss. Rodent neurons with ion channel loss-of-function appear to, therefore, be significantly more resistant to neurodegeneration compared to human neurons. Fundamental differences in susceptibility of human and rodent cerebellar neurons in ataxia-causing channelopathies must therefore be present. In this review, we explore the properties of human neurons that may contribute to their vulnerability to cerebellar degeneration secondary to ion channel loss-of-function mutations. We present a model taking into account the known allometric scaling of neuronal ion channel density in humans and other mammals that may explain the preferential vulnerability of human cerebellar neurons to degeneration in ataxia-causing channelopathies. We also speculate on the vulnerability of cerebellar neurons to degeneration in mouse models of spinocerebellar ataxia (SCA) where ion channel transcript dysregulation has recently been implicated in disease pathogenesis.

## Introduction

Ion channels play a central role in human health and disease. Historically, mutations in ion channel genes have been associated with episodic neurological disorders, such as episodic ataxia, familial migraine, and seizure disorders (Russell et al., 2013). Recent advances using next-generation sequencing approaches have recognized that ion channels are key contributors to pathology in a variety of neurological disorders, even when a ion channel gene mutation is not the primary driver of disease (Bushart and Shakkottai, 2019). These include the spinocerebellar ataxias (SCAs), a group of autosomal-dominant neurodegenerative disorders affecting primarily the cerebellum and its associated pathways. In marked contrast to the lack of neurodegeneration in episodic disorders that result from mutant ion channels, mutations or changes in expression of ion channels produce prominent cerebellar degeneration in human SCAs, suggesting a causal role for ion channel dysfunction in the degenerative process. However, in many animal models of channelopathy, structural changes are often absent. While this may reflect fundamental challenges of modeling human disease in murine models, it may also reflect a unique vulnerability of human neurons to ion channel dysfunction.

Many of the ion channel genes that cause known channelopathies are expressed widely, and in overlapping patterns, throughout the central nervous system. Neurons are highly specialized in their ability to process information, with different neuronal subtypes requiring ion channels that possess specific voltage or temporal properties to maintain normal function. These restraints could make neurons susceptible to perturbations in function, or to neurodegeneration, when they harbor mutant or inactive ion channels. It is hypothesized that overlapping properties and expression of ion channels with similar biophysical properties, which is termed “degeneracy,” might allow neurons to retain relatively normal function when individual ion channels are lost. This reflects a potential of ion channels with overlapping roles and expression to compensate for other ion channels that undergo a loss-of-function mutation (Goaillard and Marder, 2021). This process of degeneracy and compensation appears to be a plausible mechanism of resistance to neurodegeneration secondary to ion channel loss-of-function in mice. In humans, however, ion channel gene mutations that result even in haploinsufficiency can have profound behavioral and neurodegenerative consequences. This suggests that the same rules of degeneracy and compensation may not apply equally to mice and humans, and that fundamental differences in the vulnerability of human and mouse neurons may exist for the channelopathies.

In addition to progressive motor impairment, a hallmark of the ataxia-causing channelopathies in humans is cerebellar atrophy or a loss of cerebellar volume on brain imaging. Cerebellar atrophy is most commonly associated with the loss of cerebellar Purkinje neurons. Purkinje neurons are thought to be vulnerable to dysfunction and degeneration due to several of their unique features, including a high autonomous firing rate that requires the coordinated action of many different ion channels (Bushart and Shakkottai, 2019). Remarkably, ion channel loss-of-function has also been identified in the autosomal dominant cerebellar ataxias that are not directly caused by mutations in ion channel genes. Ataxia-causing loss-of-function mutations in ion channel genes in humans were initially described to solely produce a late onset, slowly progressive phenotype. More recently identified ion channel gene mutations can, however, produce an early onset phenotype with progressive cerebellar atrophy in the first and second decades of life (see below). Despite clear structural and functional consequences of channelopathies in human neurons, loss of cerebellar Purkinje neurons is not a prominent pathological feature in ion channel knock-out mice. The lack of neurodegeneration is particularly puzzling in ion channel knock-out mice where mutation in the respective disease-causing ion channel gene produces an early onset degenerative phenotype in humans. For the purposes of this review, we define neurodegeneration as is conventional for human disease; namely, either identification of frank loss of cerebellar neurons in autopsy samples or evidence of a macroscopic reduction in cerebellar volume with brain imaging, or both. In human disease, loss of cerebellar brain volume or atrophy is associated with loss of cerebellar Purkinje neurons at autopsy. The current review will discuss vulnerability of human neurons in relation to murine Purkinje neurons regarding degeneration due to mutations in ion channel genes. In addition, we will speculate on the relative resistance to degeneration in mouse models of SCA where ion channel transcript dysregulation, rather than a mutation in an ion channel gene, is associated with disease.

## Membrane Characteristics of Neurons in Human and Other Mammalian Species Used to Model Human Disease

In recent years, knowledge of human neuron membrane physiology has grown significantly with the use of resected cortical tissue. Tissue that is resected for several neurosurgical indications, including temporal lobe epilepsy and tumor, has been successfully used in preparations for patch-clamp recording. Tissue resections generally contain neurons that are representative of healthy human cortical neurons *in vivo*. This process has allowed researchers to make direct comparisons of active and passive membrane properties between neurons from human and model species, along with assessing structure-function relationships in human neurons. From these studies, is has become clear that human neurons are not simply “scaled-up” versions of neurons from other mammalian species; rather, they possess unique structural and functional properties (Mohan et al., 2015; Deitcher et al., 2017; Beaulieu-Laroche et al., 2021).

Several studies have highlighted clear differences between the input-output properties of human cortical neurons and those of model species. A comparative study of layer 5 (L5) cortical pyramidal neurons across multiple mammalian species demonstrated that human neurons diverge from a conserved electrophysiological pattern that is observed in other mammals (Beaulieu-Laroche et al., 2021). As cell size increases, with humans possessing the largest somatic size in L5 cortical pyramidal neurons, ionic conductance generally increases proportionally. However, human neurons break this pattern, exhibiting a much smaller conductance than would be predicted (Beaulieu-Laroche et al., 2021). For example, human cortical L5 neuronal somata are located at a fourfold greater depth (the depth of the soma is correlated with membrane surface area) than corresponding mouse neurons. In spite of being approximately fourfold larger than mouse neurons, the peak voltage-gated potassium current and HCN current in distal dendritic and somatic outside-out membrane patches in human neurons is similar in amplitude to that of mouse neurons. This suggests that the density of these ion channels is approximately fourfold lower than what would be predicted in humans based on the allometric scaling of these conductances in other mammalian species (Beaulieu-Laroche et al., 2021). The human cortex also contains a much higher neuronal density than predicted for the same degree of expansion in brain size from non-human primates, as compared to other mammals (Herculano-Houzel, 2009, 2012). Additionally, human neurons likely utilize distinct sets of ion channels alongside differences in passive membrane properties, including a specific membrane capacitance that is 50% lower than predicted (Eyal et al., 2016). Human cortical neurons appear to rely more heavily on HCN channels, which generate a subthreshold cation current that can greatly influence signal integration, than mouse cortical neurons (Kalmbach et al., 2018). Other ion channels have not yet been studied in great detail, but differences in somatic and dendritic spike morphology across species (Beaulieu-Laroche et al., 2021) suggest that human neurons likely place emphasis on a subset of ion channels that may be distinct from other species.

Significant differences in dendrite structure and function are also present between human cortical neurons and those from other species. Human L2–L3 cortical pyramidal neurons are much longer and have more complex branching than mouse cortical neurons (Mohan et al., 2015; Deitcher et al., 2017). Functionally, human L2–L3 neurons appear capable of producing graded calcium-mediated action potentials, which theoretically allows a much more dynamic encoding of synaptic input than simple all-or-none spikes (Gidon et al., 2020). This type of graded dendritic spike has not been described in other species, suggesting divergent function in human neurons. In human L5 cortical pyramidal neurons, increased dendritic volume and reduced dendritic ion channel density contribute to an increased input resistance and enhanced somato-dendritic coupling compared to mouse (Beaulieu-Laroche et al., 2018, 2021). In the context of low specific membrane capacitance, which has been proposed to enhance the filtering capacity of human cortical dendrites (Eyal et al., 2016), these features would alter the structure-function relationship in human dendrites. Therefore, dendritic signaling appears to be a major driver of the unique computational features of human neurons.

At present, no physiological data is available from resected human cerebellar tissue. However, a comparative study has shown that human cerebellar neuron density is higher than would be predicted by scaling to other mammals (Herculano-Houzel et al., 2015), a relationship that is also seen in human cortical neurons (Herculano-Houzel, 2009). This raises the possibility that the allometric structure-function relationship of L5 cortical pyramidal neurons that is different in humans (Beaulieu-Laroche et al., 2021) might also extend to cerebellar neurons. If this holds true, a lower-than-predicted current density in human cerebellar neurons might underlie a fundamental susceptibility to perturbances in ion channel function. In turn, further reduced current density upon channelopathy may drive structural changes in an attempt to preserve function, as has been proposed to be a mechanism of neurodegeneration in a mouse model of SCA (Dell’Orco et al., 2015).

## Ataxia-Causing Channelopathies Due to Loss of Ion Channel Function in Humans and Mice

In humans, a number of ion channelopathies cause various forms of episodic ataxia and autosomal dominant SCA. Heterozygous mutations in ion channel genes are sufficient to cause disease, and haploinsufficiency of the causative ion channel is the putative mechanism in many cases. In the last decade, several ion channelopathies, particularly in potassium channel genes, have been identified to result in widespread neurodegeneration of cerebellar and sometimes cortical and other deep brain structures. These disorders are often associated with behavioral alterations and motor dysfunction, although clinical heterogeneity is observed. However, when equivalent ion channel haploinsufficiency or even full channel knockout models in mice are generated, murine models are often resistant to motor and morphological alterations (Table 1). This section will highlight the clinical and morphological sequelae of ataxia-causing channelopathies in humans in comparison to what is observed in rodents.

**TABLE 1:** Phenotypic and structural alterations in human and mouse ion channel gene loss-of-function mutations.

Gene name (channel name)	Citations	Observed phenotype in human	Structural changes in humans (on brain imaging unless otherwise specified)	Observed phenotype in mouse	Structural changes in mouse
*KCNMA1* (BK, K_Ca_1.1)	Meredith et al., 2004; Sausbier et al., 2004; Liang et al., 2019; Du et al., 2020; Liang et al., 2022	Liang–Wang syndrome: Death in early onset cases due to a multiple visceral malformation syndrome, craniofacial dysmorphism Developmental delay with speech delay, and ataxia in milder cases	Progressive cerebellar atrophy that is variable but sometimes severe Mild cerebral atrophy Thin corpus callosum	Tremor Abnormal gait Decreased time to fall (rotarod) Overactive bladder	None noted in *Kcnma1* knockout
*KCNN2* (SK, K_Ca_2.2)	Callizot et al., 2001; Shakkottai et al., 2004; Szatanik et al., 2008; Mochel et al., 2020	Developmental delay Early onset cerebellar ataxia Extrapyramidal symptoms	Diffuse periventricular white matter changes Cerebellar atrophy (Mochel personal communication in initially identified case)	Frissonnant: Decreased time to fall (rotarod) Decreased locomotor activity Tremor *Kcnn2* knockout: Tremor *Kcnn2* dominant-negative suppression: Decreased time to fall (rotarod) Gait ataxia Tremor	None noted in any model
*KCNC3* (K_v_3.3)	Herman-Bert et al., 2000; Espinosa et al., 2001; Waters et al., 2006; Hurlock et al., 2008	Childhood-onset ataxia or late-onset ataxia Cognitive delay	Global cerebellar volume loss	Decreased time to fall (rotarod) Myoclonus Ethanol hypersensitivity	None noted in *Kcnc3* knockout
*KCND3* (K_v_4.3)	Niwa et al., 2008; Duarri et al., 2012; Lee et al., 2012	Slowly progressive cerebellar ataxia Urinary urgency, incontinence	Mild cerebellar atrophy, Purkinje neuron loss at autopsy (one case)	No neurologic deficits	None noted in *Kcnd3* knockout
*ITPR1* (IP3 receptor 1)	Matsumoto et al., 1996; Miyoshi et al., 2001; van de Leemput et al., 2007; Iwaki et al., 2008; Sugawara et al., 2013	Cerebellar ataxia Head tremor	Cerebellar atrophy without brainstem involvement	Early death in global knockout with ataxia and seizures Cerebellar ataxia in Purkinje neuron specific knockout	No structural changes noted Increase in dendritic spine density with simplification of dendrites in Purkinje neuron specific deletion
*CACNA1G* (Ca_v_3.1)	Chemin et al., 2018; Coutelier et al., 2015; Morino et al., 2015; Kimura et al., 2017; Li et al., 2018; Hashiguchi et al., 2019; Barresi et al., 2020	Infantile or childhood-onset cerebellar ataxia, global developmental delay in gain-of-function mutations Late onset ataxia with loss-of-function mutations	Childhood-onset cerebellar atrophy Cerebellar atrophy with Purkinje neuron loss at autopsy	Decreased time to fall (rotarod) Increased gait width	Late Purkinje cell loss in loss-of-function mutation
*CACNA* (Ca_v_2.1)	Ophoff et al., 1996; Zhuchenko et al., 1997; Jun et al., 1999; Fletcher et al., 2001; Pietrobon, 2002; Du et al., 2013; Reinson et al., 2016; Jen and Wan, 2018	Episodic ataxia type 2: episodes that respond to acetazolamide and spontaneously remit in later life Allelic with Familial hemiplegic and Spinocerebellar ataxia type 6	Variable and selective atrophy of the cerebellar vermis in some cases late in life	Low body weight Decreased lifespan Decreased time to fall (rotarod) Ataxia Seizure	Early Purkinje, granule and Golgi cell loss

## Gene: *KCNMA1*, Ion Channel: BK or Large-Conductance Calcium-Activated Potassium Channel

*KCNMA1* encodes the large conductance calcium-activated potassium channel, also known as the BK or K_Ca_1.1 channel. Mutations in *KCNMA1* cause Liang–Wang syndrome, comprised of developmental delay, cerebellar ataxia, and variable neurological features including seizures and dystonia (Liang et al., 2019), with visceral malformations and death in infancy in the most severe cases. Variable neurodegeneration is also noted on MRI, with early onset of progressive atrophy of the cerebellar hemispheres and vermis in the first and seconds decades of life in multiple patients (Liang et al., 2019, 2022; Du et al., 2020). In a heterologous expression system, BK channel mutations result in loss of BK channel current, with postulated haploinsufficiency and dominant-negative mechanisms (Liang et al., 2019, 2022; Du et al., 2020).

The G354S *KCNMA1* mutation introduced systemically using AAV9 results in gait dysfunction in mice (Du et al., 2020). No neurodegeneration was reported in AAV9-G354S *KCNMA1* mice (Du et al., 2020). G354S *KCNMA1* has been hypothesized to induce mitochondrial dysfunction as the mechanism for disease. Immunogold labeling for *Kcnma1* in mice indicates, however, that BK channels in mice are mostly located at the plasma membrane rather than intracellularly (Kaufmann et al., 2009). This suggests that BK channel function at the plasma membrane is likely a primary mechanism of motor impairment and cerebellar degeneration in humans, but that mice are resistant to BK channel-dysfunction mediated neurodegeneration. In separate mouse models, complete knockout of *Kcnma1* produces profound ataxia and Purkinje neuron firing impairment in mice, yet cerebellar degeneration is not observed (Meredith et al., 2004; Sausbier et al., 2004). Together, these studies suggest that BK channels play a very important role for cerebellar function in both humans and mice, but that humans are much more vulnerable to both motor impairment (haploinsufficiency in humans vs. complete null in mice) and cerebellar neurodegeneration due to BK channel mutations.

## Gene: *KCNN2*, Ion Channel: SK2 or Small-Conductance Calcium-Activated Potassium Channel 2

*KCNN2* encodes the small conductance calcium-activated potassium channel, known as SK2 or K_Ca_2.2. Recently, point mutations in *KCNN2* have been described to result in a spectrum of neurodevelopmental movement disorders (Mochel et al., 2020). Patients generally experience intellectual disability, motor delay, and language delay, along with behavioral comorbidities. Additionally, movement disorders with early age-of-onset are present in many patients, including cerebellar ataxia and extrapyramidal symptoms. White matter abnormalities were observed in three patients out of six for whom MRI data was available, while cerebellar atrophy is also observed in at least one case (Mochel et al., 2020) (Mochel personal communication). The SK2 channel variants are postulated to cause disease through haploinsufficiency.

Interestingly, spontaneous *Kcnn2* mutations have been identified in several mouse strains (Callizot et al., 2001; Szatanik et al., 2008). These mouse strains show tremor and behavioral dysfunction but are not reported to undergo neurodegeneration. Additionally, a mouse model that utilizes a dominant-negative SK channel construct to functionally silence SK channels in the brain also does not display neurodegeneration despite demonstrating prominent motor impairment (Shakkottai et al., 2004). Overall, SK channel loss-of-function appears to induce cerebellar atrophy (in one case) and structural changes in human white matter but does not induce neurodegeneration in mice.

## Gene: *KCNC3*, Ion Channel: Kv3.3 or Voltage-Gated Potassium Channel of the Kv3 Family

*KCNC3* encodes a member of the voltage-gated potassium channel in the Kv3 family. Loss-of-function mutations in *KCNC3* produces a neurological disorder ranging from abnormal neurodevelopment to an adult-onset neurodegenerative disorder (Waters et al., 2006), now termed SCA13. Cerebellar atrophy and slow disease progression, even in early onset cases, is a prominent feature of disease. A putative dominant negative loss-of-function of this ion channel that is enriched in the cerebellum is the postulated mechanism of disease.

Kv3.3 null mice were initially described to have no overt phenotype (Espinosa et al., 2001). Subsequent to the identification of SCA13 due to *KCNC3* mutations, Kv3.3 null mice were identified to have a mild motor phenotype, with primarily increased lateral deviation while ambulating and foot slips when traversing a narrow beam (Hurlock et al., 2008). Nevertheless, no progressive or degenerative phenotype is seen even in mice in which both Kv3.3 and the related Kv3.1 channel are deleted, despite a much more severe ataxic phenotype in Kv3.1/Kv3.3 double null mice compared to Kv3.3 null mice (Espinosa et al., 2001).

## Gene: *KCND3*, Ion Channel: Kv4.3 or Voltage-Gated Potassium Channel of the Kv4 Family

*KCND3* encodes a voltage-gated potassium channel of the Kv4 family. Heterozygous loss-of-function mutations in Kv4.3 results in a late-onset SCA that was identified independently by two separate groups, and is now termed SCA19/22 (Duarri et al., 2012; Lee et al., 2012). Slowly progressive ataxia in mid-life with cerebellar atrophy is characteristic of disease. Cerebellar Purkinje neuron loss is seen in the one described individual with SCA19 who has gone to autopsy (Duarri et al., 2012).

Kv4.3 null mice are described to be indistinguishable from wild-type mice (Niwa et al., 2008). Recording from cardiac ventricular myocytes, an area where Kv4.3 is highly expressed, was also found to be normal *via* cardiac electrophysiology (Niwa et al., 2008).

## Gene: *ITPR1*, Ion Channel: IP3 Receptor 1

*ITPR1* encodes an intracellular calcium channel, the IP3 receptor, that is highly enriched in the cerebellum. Deletions in one copy of the *ITPR1* gene results in autosomal dominant SCA15/16 (van de Leemput et al., 2007; Iwaki et al., 2008), suggesting that haploinsufficiency is sufficient to cause human disease. SCA15/16 presents as slowly progressive, relatively pure cerebellar ataxia with age of onset in the 4th decade of life. Prominent cerebellar atrophy without brainstem involvement is a hallmark of disease (Miyoshi et al., 2001).

Homozygous deletion of *Itpr1* in mice results in death *in utero*, with surviving mice displaying ataxia and epileptic seizures with death by the weaning period (Matsumoto et al., 1996). Although brains of *Itpr1* null mice are smaller, they are histologically indistinguishable from wild-type mice (Matsumoto et al., 1996). Heterozygous *Itpr1* deficient mice are described to have no obvious defects (Matsumoto et al., 1996). Mice with a Purkinje neuron specific deletion of *Itpr1* display progressive ataxia. These mice have normal overall cerebellar morphology, including the organization of the cerebellar cortical layer and Purkinje neuron counts at 10 weeks, but display fewer Purkinje neuron dendritic branch points and a greater spine density (Sugawara et al., 2013). This suggests that *Itpr1* plays a functional role in the cerebellum in both mice and humans, but only humans exhibit neurodegeneration secondary to loss of the type 1 IP3 receptor.

## Gene: *CACNA1G*, Ion Channel: CaV3.1 or T-Type Voltage-Gated Calcium Channel

*CACNA1G* encodes Ca_V_3.1, a T-type voltage-gated calcium channel. Putative gain-of-function, *de novo* point mutations in *CACNA1G* are associated with a neurodevelopmental disorder that includes motor delay, cerebellar ataxia, apraxia, strabismus, and dysmorphic features (Chemin et al., 2018; Barresi et al., 2020). Extensive cerebellar atrophy, mainly in the vermis but sometimes globally, is observed in these patients, while brainstem and cerebral cortex are largely unaffected (Chemin et al., 2018; Barresi et al., 2020). On the other hand, a loss-of-function mutation, p.Arg1715His, causes the autosomal-dominant spinocerebellar ataxia SCA42 (Coutelier et al., 2015; Morino et al., 2015; Kimura et al., 2017). SCA42 results in onset of ataxia and cerebellar atrophy in mid-life and a recurrent p.Arg1715His mutation in Cav3.1 in reported in individuals in France and Japan. An additional mutation in individuals in a family in China with onset of ataxia in mid-life has been reported due to a p.Met1574Lys mutation in Cav3.1 (Li et al., 2018).

In a mouse model of SCA42, knock-in of the mutation analogous to p.Arg1715His (p.Arg1723His in mice) induces motor impairment at 10 weeks that is stable. Mild Purkinje neuron loss is not evident until 50 weeks, without progression of motor impairment (Hashiguchi et al., 2019). In the case of *CACNA1G*, a mouse model of disease recapitulates some of the motor impairment and neurodegeneration observed in human disease, although there is a disconnect, as seen in other channelopathies, between the onset and degree of motor impairment and cerebellar degeneration.

## Gene: *CACNA1A*, Ion Channel: CaV2.1 or P/Q Type Voltage-Gated Calcium Channel

*CACNA1A* has emerged as an important disease gene that causes a range of episodic disorders and autosomal dominant SCA. *CACNA1A* encodes Ca_V_2.1, a P/Q type voltage-gated calcium channel that is enriched in presynaptic terminals and in Purkinje neuron cell bodies. *CACNA1A* mutations are the cause of episodic ataxia type 2 (EA2) (Ophoff et al., 1996), familial hemiplegic migraine (FHM) (Ophoff et al., 1996), and spinocerebellar ataxia type 6 (SCA6), a CAG triplet repeat expansion disorder (Zhuchenko et al., 1997). Progressive cerebellar neurodegeneration and motor impairment are present in SCA6, although the mechanism of disease remains unclear. While loss-of-function of Ca_V_2.1 may underlie disease, growing evidence suggests that α1ACT, a separately translated portion of the channel that contains its C-terminal fragment, plays an important role in the disease process (Du et al., 2013). Therefore, it is difficult to speculate on the role of ion channel loss-of-function vs. α1ACT C-terminal toxicity specifically in SCA6. However, in EA2, point mutations in *CACNA1A* lead to early onset of episodes of unsteady gait, slurred speech, and incoordination, in infancy or childhood and sometimes accompanied by progressive ataxia between episodes and variable late cerebellar atrophy (Jen and Wan, 2018). Biallelic *CACNA1A* mutations cause an epileptic encephalopathy, with developmental delay and progressive cerebral and cerebellar atrophy (Reinson et al., 2016).

In mice, Purkinje cell-specific knockout of *Cacna1a* induces motor impairment and causes Purkinje neuron loss beginning at post-natal day 30 (Todorov et al., 2012). A number of other recessive loss-of-function mutations in *Cacna1a* (termed *tottering*, *leaner*, *rolling Nagoya*, and *rocker*) produce a phenotype of ataxia, seizures, and dystonia/dyskinesia in mice. In the *leaner* cerebellum, there is an early and significant loss of cerebellar granule, Golgi, and Purkinje cells (Pietrobon, 2002). *Cacna1a* null mice exhibit a phenotype of ataxia and degeneration of cerebellar neurons, including Purkinje cells, (Jun et al., 1999; Fletcher et al., 2001) similar to *leaner* mice. Thus, *Cacna1a* mutations may be the exception to the general observation that loss-of-function mutations in ion channel genes do not produce degeneration in mouse models. Paradoxically, however, the degenerative phenotype resulting from *Cacna1a* loss-of-function is more prominent in mice as compared to humans where it causes primarily an episodic disorder with variable and late onset of cerebellar atrophy.

## Ion Channel Reserve and Degeneracy as a Vulnerability Factor in Cerebellar Disease

As outlined above, human ataxia-causing channelopathies and mouse models of ion channel knockout, particularly for potassium channel genes, show diverging patterns of neurodegeneration and even a complete lack of degeneration in some mouse models. A simplistic argument could be that murine Purkinje neurons are merely more resistant to degeneration. Also, the cerebellum is not uniformly vulnerable to degeneration in human disease, and is resistant to degeneration in canonical age related human neurodegenerative disease (Liang and Carlson, 2020). The idea that mouse Purkinje neurons are more resistant to degeneration is contradicted by several rodent models of human cerebellar ataxia where Purkinje neurons are in fact very vulnerable to degeneration in both mice and humans. For example, in a mouse model of Neiman-Pick C, cerebellar Purkinje neurons undergo profound neurodegeneration, similar to human disease (Elrick et al., 2010), with an almost complete loss of neurons by post-natal week 22. Interestingly, in this study, Purkinje neuron intrinsic excitability (a marker of ion channel dysfunction) was unaffected even in the presence of marked Purkinje neuron atrophy. In marked contrast to the mutations in ion channel genes where the previously developed ion channel-knockout mice displayed no degeneration, *pcd* mice were identified over four decades ago to have loss of virtually all cerebellar Purkinje cells during the third and fourth post-natal week (Landis and Mullen, 1978). Biallelic variants of the homologous gene, *AGTPBP1*, which codes for a metallocarboxypeptidase that mediates protein deglutamylation of tubulin and non-tubulin target proteins, were recently identified to cause childhood-onset neurodegeneration with profound cerebellar atrophy (Shashi et al., 2018; Sheffer et al., 2019). It is interesting to speculate that the divergence between mouse and human Purkinje neurons in the vulnerability to degeneration is unique to cerebellar disorders of ion channel dysfunction. Rules of compensation and degeneracy may be a key difference in ion channel regulation in humans vs. mice compared to other genes. This is supported by studies in human cortical neurons where the rules of allometric scaling of ion channel density shows clear divergence between human neurons and all other species (Beaulieu-Laroche et al., 2021). Human cerebellar neurons may, therefore, be particularly vulnerable to disease processes that reduce ion channel density, including channelopathies.

In cases of ion channel loss-of-function or knockout, degeneracy or substitution with a different ion channel may compensate for loss of an ion channel. Increased functional reliance on an ion channel that has a similar, but incompletely, overlapping function to the channel that has become non-functional is a mechanism to preserve function. This is perhaps most clearly observed in a knockout mouse model of *Kcnc* channels (K_v_3 family members), where genetic knockout of *Kcnc3* fails to induce phenotypic features of disease if the compensatory gene *Kcnc1* is present (Hurlock et al., 2009). However, when *Kcnc1* is knocked out alongside *Kcnc3*, electrophysiologic dysfunction and motor impairment both appear. This is likely due to the similar functional properties of K_v_3.1 and K_v_3.3 that enable full compensation of function when K_v_3.3 is lost. Interestingly, this same mechanism of compensation does not appear to prevent phenotypic features of disease in humans, as *KCNC3* mutations cause SCA13, in which cerebellar neurodegeneration and progressive motor dysfunction are observed (Waters et al., 2006). In other experimental models, ion channel compensation has been hypothesized to involve more functionally distinct channels that preserve a level of neuronal function, although function may become slightly altered (Drion et al., 2015; Goaillard and Marder, 2021).

**FIGURE 1:**
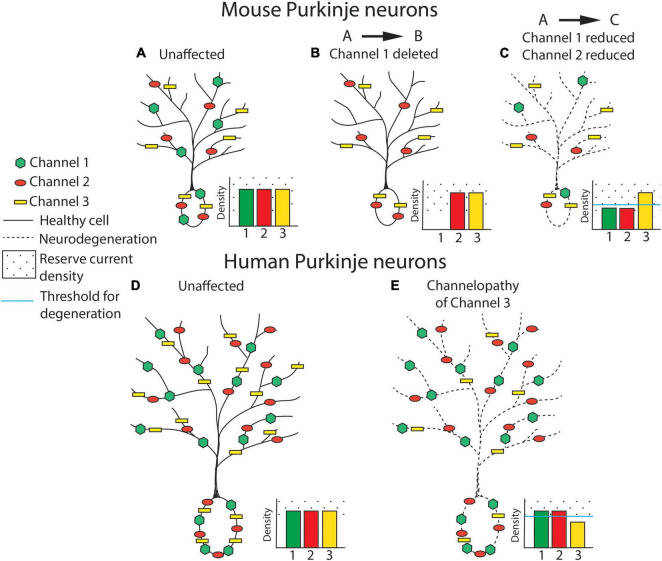
Ion channel loss differentially influences cerebellar neurodegeneration in mice and humans. Ion channel density is represented in cerebellar Purkinje neurons from mouse **(A–C)** and human **(D,E)**. In this basic model, ion channel types are hypothesized to be relatively equivalent for their influence on neurodegeneration and are therefore represented generically as Channel 1 (green hexagon), Channel 2 (red circle), and Channel 3 (yellow rectangle). For each panel, density of expression for each ion channel is represented in a bar plot on the inset of each neuron, with the threshold of channel loss needed to produce neurodegeneration represented by a solid blue line and ion channel “reserve density” noted with a dotted background. Note that the level of reserve ion channel density to produce channel dysfunction is higher in mice for each channel, and that the baseline ion channel density is similar in human and mouse neurons. **(A)** An unaffected wild-type mouse neuron is represented with normal expression of channels 1, 2, and 3. **(B)** Full knockout of an individual ion channel (channel 1) produces no Purkinje neuron degeneration in mice, possibly due to increased function of overlapping or compensatory ion channels. **(C)** Partial reduction of multiple ion channels within the same excitability pathway, as has been observed in mouse models of spinocerebellar ataxia (SCA), is sufficient to produce Purkinje neuron atrophy and neurodegeneration despite each individual channel remaining within a “reserve” level of expression. Degeneration is represented as a cell membrane with a dashed line. **(D)** An unaffected, normal human neuron is represented with normal expression of channels 1, 2, and 3. The density of ion channels is the same as in mouse neurons in spite of a considerable increase in cell size. **(E)** In humans, haploinsufficiency of a single ion channel is sufficient to induce clinical symptoms and Purkinje neuron degeneration. Degeneration is represented as a dashed cell membrane.

An alternative compensatory mechanism to the ion channel substitution/degeneracy described above is compensation for partial loss of ion channels through functional reserve. The baseline ion channel density, and how much ion channel exists in excess of what is needed, may predicate how much ion channel protein must be lost before there is impairment in function. Differences in ion channel density may explain differences in vulnerability of human and mouse neurons to neurodegeneration in channelopathies. This concept is outlined in Figure 1. A reduced baseline ion channel density in human cerebellar neurons, as has been observed in human cortical neurons (Beaulieu-Laroche et al., 2021), may make them more sensitive to perturbations in ion channel expression or function. In mice, reduced ion channel expression may be tolerated if it does not surpass the level of ion channel reserve needed to maintain normal function. Even in cases of full ion channel knockout, a compensatory ion channel may then act to preserve normal neuronal function.

Despite lack of neurodegeneration in many mouse models of ion channel knockout, cerebellar neurodegeneration is observed in mouse models of SCA that model human SCAs due to a polyglutamine encoding, CAG repeat-expansion in the respective disease genes (so-called polyglutamine ataxia). In mouse models of SCA1 (Dell’Orco et al., 2015; Bushart et al., 2021), SCA2 (Kasumu et al., 2012; Hansen et al., 2013; Dell’Orco et al., 2017), SCA3 (Shakkottai et al., 2011), SCA6 (Jayabal et al., 2016), and SCA7 (Stoyas et al., 2020), onset of motor impairment is concurrent with Purkinje neuron intrinsic firing abnormalities and in many cases long precedes the onset of Purkinje neuron loss. In these diseases, where the respective disease-causing mutations do not reside in ion channels, transcriptional alterations are the likely driver of disease. Remarkably, a reduction in transcript levels of ion channels that drive electrophysiologic dysfunction are shared across several of these disorders (Chopra et al., 2020; Stoyas et al., 2020). In murine models of disease, reduced transcripts for *Kcnma1*, *Cacna1g*, *Trpc3*, and *Itpr1* are seen (Chopra et al., 2020). Interestingly, pharmacologic inhibition of any of these individual ion channels fails to induce spiking abnormalities in wild-type Purkinje neurons, yet inhibition of two or more of these channels simultaneously is sufficient to induce the changes in Purkinje neuron spiking that are observed in mouse models of disease (Chopra et al., 2020; Stoyas et al., 2020). Restoring ion channel expression or function attenuates neurodegeneration in mouse models of SCA1 (Dell’Orco et al., 2015; Chopra et al., 2018) and SCA2 (Kasumu et al., 2012), suggesting that a reduction in ion channel transcripts is a driver of neurodegeneration. If ion channel dysfunction is a driver of neurodegeneration in these disorders, is this an apparent contradiction to the lack of neurodegeneration that is observed in mouse knock-out models of individual ion channel genes? It is possible that by affecting several ion channel transcripts simultaneously, the redundancy and ability to compensate for loss of individual ion channels in mouse Purkinje neurons is now no longer possible. Neurodegeneration in the polyglutamine ataxias in rodent models of disease requires very high overexpression of the mutant protein in transgenic models, or repeat expansion lengths far greater than what is normally observed in human disease in knock-in models of disease. Consistent with the hypothesis that ion channels may be an important driver of neurodegeneration in the polyglutamine ataxias, the degree of binding of the mutant protein in SCA1 is polyglutamine length-dependent, and simultaneously reduces transcripts of several of the channels involved in ataxia-causing channelopathy (Hansen et al., 2013). This may explain the need for overexpression of disease-causing polyglutamine expanded protein to cause sufficient ion channel transcript suppression, therefore overcoming greater ion channel reserve in mice for individual ion channels.

Is the allometric scaling and vulnerability to degeneration of human cerebellar neurons unique to ion channel dysfunction? While there are instances of Purkinje degeneration that occurs in a similar fashion both mice and in humans as outlined above, there are other causes of ataxia, such as in disorders of DNA repair, which also appear to have a disconnect between degeneration that is profound in human disease yet is absent in mouse knockout models. In models of ataxia telangiectasia and ataxia with oculomotor apraxia type 1, complete knockout of the proteins ATM (Lavin, 2013) and APTX (Ahel et al., 2006), respectively, fails to produce motor impairment, cerebellar atrophy, or Purkinje neuron loss (Lavin, 2013; Perez et al., 2021). In a recently developed mouse model, a combination of a biallelic human *ATM* mutation and knockout of *APTX* produces motor impairment and cerebellar volume loss without loss of Purkinje neurons by post-natal day 400. Surprisingly, the progressive motor impairment is associated with slowed Purkinje neuron firing in this model of ataxia-telangiectasia reminiscent of the changes in firing seen in the models of SCA described previously. The ionic basis for the reduction in Purkinje neuron firing in this model of ataxia-telangiectasia remains unexplored. Alterations in Purkinje neuron spiking are also seen in a different DNA break repair disorder. *Xrcc1* knockout mice, a model for an autosomal recessive cerebellar ataxia due to biallelic loss-of-function mutations in the homologous human gene, display slowed Purkinje neuron spiking (Hoch et al., 2017). This somewhat unexpected finding of altered Purkinje neuron spiking in these models is consistent with the idea that even in disorders of DNA break-repair, the underlying mechanism for degeneration may in fact be secondary to ion channel dysregulation. While there may be other biological processes other than ion channel density that do not scale with the increase in human neuron size, ion channel density is clearly worth investigating as a unique vulnerability factor for degeneration of human cerebellar neurons.

The mechanism for how ion channel dysfunction leads to neurodegeneration in cerebellar disorders remains unclear. A plausible hypothesis is that it is related to aberrant calcium homeostasis. Purkinje neurons are unusual in that the inward calcium current is completely masked by an outward potassium current (Raman and Bean, 1999). Rapid clearance of intracellular calcium is needed in the interspike interval of Purkinje neurons that exhibit high rates of firing (Fierro et al., 1998). Clearance of cellular calcium requires plasma membrane calcium pumps and the sodium-calcium exchanger, also located on the plasma membrane (Fierro et al., 1998). Since the volume of the cell that is cleared of calcium scales as an order of magnitude greater than the surface area of the cell, it may explain the observed increase in ion channel density with increased cell size in most mammalian species. Humans appear to not have sufficient reserve for the increase in calcium clearance mechanisms with the increase in cell size, at least in cortical neurons.

## Suitability of Mouse Models for Ataxia-Causing Channelopathies

This review presents an argument that ion channel loss-of-function produces distinct patterns of neurodegeneration in humans that are not replicated in mouse models of disease. In complex neurodegenerative conditions where ion channel mutations are not the primary drivers of disease, such as in the polyglutamine SCAs, widespread transcriptional alterations appear to be associated with disease. In these cases, a shared reduction in transcripts of several ion channel genes contributes to neurodegeneration. However, neurodegeneration is not observed in any potassium channel knockout mice. As outlined above, single ion channel deletion appears to be well compensated in mouse Purkinje neurons due to ion channel reserve and overlapping functional roles of similar ion channel genes. While ion channel knockout models have been valuable for understanding the major electrophysiological and behavioral roles of these ion channels, they do not model the neurodegeneration seen in human ataxia causing channelopathies.

## Conclusion

Human ion channelopathies are closely associated with cerebellar neurodegeneration. Despite this association, ion channel knockout in mice rarely causes neurodegeneration that mirrors human cerebellar disease. This review has presented key differences in cortical neuron function between human and model species. If true also of cerebellar neurons, it suggests that fundamental differences in cerebellar neuron function may make human cerebellar neurons more vulnerable to perturbations in ion channel function. In cases where there is a disconnect in cerebellar degeneration between mouse models and human disease, it may be worth looking for ion channel dysfunction as the basis for cerebellar neurodegeneration.

## Author Contributions

DB and VS wrote and edited the manuscript. Both authors contributed to the article and approved the submitted version.

## Conflict of Interest

The authors declare that the research was conducted in the absence of any commercial or financial relationships that could be construed as a potential conflict of interest.

## Publisher’s Note

All claims expressed in this article are solely those of the authors and do not necessarily represent those of their affiliated organizations, or those of the publisher, the editors and the reviewers. Any product that may be evaluated in this article, or claim that may be made by its manufacturer, is not guaranteed or endorsed by the publisher.
